# 

**Published:** 1916-12-30

**Authors:** 


### DORSET ESSAY COMPETITION.

The trustees of the Cecil and Mansell-Pleydell medals, in connection with the Dorset Field Club, announce that the Cecil medal and prize of £10, now competed for every other year, will be awarded in May next for the best paper on "The More Recent Applications of Electricity in the Present War, especially in the Treatment of Diseases and Wounds arising Therefrom." This is a timely and very interesting subject for research. The competition will be open to persons between the ages of seventeen and thirty-five on May 1, 1917, born in Dorset or who have resided in the county for not less than one year between May 1, 1915, and May 1, 1917. Mr. H. Ponney, of the *Dorset County Chronicle* Office, Dorchester, will be pleased to give full particulars.

### Gifts to Hospitals.

Lord Portman and Lord Barnard have sent gifts of pheasants to the Royal Hospital for Incurables, Putney Heath.

Mrs. E. H. Burchmore, of St. Albans, who died on October 29, has left £250 to St. Albans Hospital and £200 to the London Orphan Asylum.

Sir John Tatem has provided a recreation hut, to accommodate 200 men, at the Cardiff Mental Hospital, a gift which is much appreciated by the committee and the convalescents.

Miss Mary Pilcher, of Hertford Street, Mayfair, W., left £500 to the British Home for Incurables, Streatham; £200 to St. Andrew's Convalescent Home, Folkestone; and £200 to the Royal Orthopædic Hospital.

Mr. James Woodward, of Moseley, left £50 each to the National Lifeboat Institution, the Middlemore Emigration Home, the General Hospital and the Queen's Hospital at Birmingham, and the Children's Hospital for the Blind at Edgbaston.

Colonel Thomas Edgar Jobling, of Bebside Hall, Northumberland, left the following, among other bequests, to be paid on the death of his sister : £2,000 each to the Newcastle Infirmary and the Fleming Memorial Hospital; £1,000 each to the Northern Counties' Deaf and Dumb Institute, Newcastle Hospital for Incurables, Royal Victoria Blind Asylum, Newcastle, and the Home for Crippled Children, Garforth.

### A NOTABLE MAYORALTY AT WIGAN.

Alderman A. S. Hilton has had the pleasure of handing to the Wigan Infirmary Board a cheque for £2,955 5s. 5d., the successful result of an effort he initiated and carried through during his mayoralty with the object of completely clearing off the debt on the new out-patients' department at the infirmary, which was erected as a memorial to King Edward VII.

RATES OF SUBSCRIPTION: Twelve months, 6/6; Six months, 4/-; Three months, 2/-; post free to any address in the United Kingdom. Abroad, Twelve months, 8/8; Six months, 5/-; Three months, 2/6, post free. THE HOSPITAL "Index" to Volume LX., April to September 1916, is now ready, price 2½d. per copy, post free. Address The Manager, "THE HOSPITAL," The Hospital Building, 28/29 Southampton Street, Strand, London, W.C.

